# A prospective study assessing agreement and reliability of a geriatric evaluation

**DOI:** 10.1186/s12877-017-0546-9

**Published:** 2017-07-19

**Authors:** Isabella Locatelli, Stéfanie Monod, Jacques Cornuz, Christophe J. Büla, Nicolas Senn

**Affiliations:** 1 Department of Ambulatory Care and Community Medicine, University of Lausanne, Lausanne, Switzerland; 2 Public Health Office, Canton de Vaud, Lausanne, Switzerland; 3 Service of Geriatric Medicine & Geriatric Rehabilitation, University of Lausanne Hospital Center, Lausanne, Switzerland; 4 Institute of Social and Preventive Medicine, University of Lausanne, Lausanne, Switzerland; 5 Institute of family medicine, Department of ambulatory care and community medicine, University of Lausanne, Lausanne, Switzerland

**Keywords:** Geriatric consultation, Reliability, Agreement, Kappa index, Intraclass correlation coefficient

## Abstract

**Background:**

The present study takes place within a geriatric program, aiming at improving the diagnosis and management of geriatric syndromes in primary care. Within this program it was of prime importance to be able to rely on a robust and reproducible geriatric consultation to use as a gold standard for evaluating a primary care brief assessment tool. The specific objective of the present study was thus assessing the agreement and reliability of a comprehensive geriatric consultation.

**Method:**

The study was conducted at the outpatient clinic of the Service of Geriatric Medicine, University of Lausanne, Switzerland. All community-dwelling older persons aged 70 years and above were eligible. Patients were excluded if they hadn’t a primary care physician, they were unable to speak French, or they were already assessed by a geriatrician within the last 12 months. A set of 9 geriatricians evaluated 20 patients. Each patient was assessed twice within a 2-month delay. Geriatric consultations were based on a structured evaluation process, leading to rating the following geriatric conditions: functional, cognitive, visual, and hearing impairment, mood disorders, risk of fall, osteoporosis, malnutrition, and urinary incontinence. Reliability and agreement estimates on each of these items were obtained using a three-way Intraclass Correlation and a three-way Observed Disagreement index. The latter allowed a decomposition of overall disagreement into disagreements due to each source of error variability (visit, rater and random).

**Results:**

Agreement ranged between 0.62 and 0.85. For most domains, geriatrician-related error variability explained an important proportion of disagreement. Reliability ranged between 0 and 0.8. It was poor/moderate for visual impairment, malnutrition and risk of fall, and good/excellent for functional/cognitive/hearing impairment, osteoporosis, incontinence and mood disorders.

**Conclusions:**

Six out of nine items of the geriatric consultation described in this study (functional/cognitive/hearing impairment, osteoporosis, incontinence and mood disorders) present a good to excellent reliability and can safely be used as a reference (gold standard) to evaluate the diagnostic performance of a primary care brief assessment tool. More objective/significant measures are needed to improve reliability of malnutrition, visual impairment, and risk of fall assessment before they can serve as a safe gold standard of a primary care tool.

**Electronic supplementary material:**

The online version of this article (doi:10.1186/s12877-017-0546-9) contains supplementary material, which is available to authorized users.

## Background

The present study aimed to describe the *reproducibility* of a structured geriatric consultation for the identification of geriatric syndromes in older patients. While geriatric consultations are standardized with the use of validated instruments and assessment protocols, the overall evaluation of the patient and the conclusions of the consultation may differ from one geriatrician to another. Moreover, physical and psychological conditions can change considerably from one moment to another in older patients. Furthermore, comprehensive consultations by geriatricians are important as they frequently serve as reference consultations for primary care physicians and other care providers, and they offer the basis for initiating management options. In Switzerland, geriatric departments of hospitals can also provide ambulatory geriatric consultations for old patients referred by GP’s. In this perspective, the current work represents a rare occasion to provide information on how reproducible a geriatric consultation is.

The present study takes place within the AGE (Active Geriatric Evaluation) program [1], which aims at improving the diagnosis and management of geriatric syndromes in primary care by developing and evaluating an adapted clinical tool (a brief assessment tool - BAT). A description of BAT can be found in Senn et al. [2]. A formal assessment of the performance of BAT needs a robust gold standard and is thus possible only once the reproducibility (agreement and reliability) of a comprehensive geriatric evaluation is proved. This is namely the aim of the present study. Indeed, if it is rather common to assess the reliability of individual tests in controlled settings, we are not aware of studies assessing the reliability of an entire geriatric clinical consultation routinely performed, which is not simply the addition of individual tests.

In order to assess reproducibility of a geriatric consultation, repeated measures for the same patient are needed. Repeated measurements can be performed either by a same rater at different time points (*test-retest* reproducibility) or by several raters at the same time point (*inter-rater* reproducibility). In both cases two different questions arise: how good is the *agreement* between visits or raters, and how *reliable* is the measurement. Both concepts represent aspects of a measurement’s reproducibility [3]. In the simple case of a binary characteristic an intuitive measure of agreement is the proportion of individuals equally classified by the two raters or at the two visits (*observed agreement*). The *observed disagreement* is the proportion of individuals differently classified. The well-known Kappa index [4] corrects the observed agreement taking into account the so-called *chance agreement*, i.e. the proportion of equally classified cases that would be obtained just by chance. Different authors have underlined that this correction makes the index to depend crucially upon the between-subject variability, which turns the Kappa into a reliability index [5–8]. Fleiss and Cohen [8] demonstrated that, in the case of a binary variable and for sufficiently large samples, the Kappa index can be approximated by the proportion of between-subject variability over the total variability: subject, visit *or* rater, and random, which defines another well-known reliability index, the *Intraclass Correlation Coefficient*, ICC [9, 10].

In clinical studies, several sources of error variability often arise when both the time and the rater are changing, i.e. several raters evaluate subjects at different time points. This is also the case of our geriatric consultation. In such kind of situations, which cannot be drowned to the simple test-retest or inter-rater case, one should consider both the time *and* the rater (besides the random) effect in the variance decomposition, so defining a *Three-Way Intraclass Correlation Coefficient* (3w-ICC) [11, 12], and a *Three-Way Observed Disagreement Index* (3w-OD). These quantities have been used in the present study in order to estimate reliability and agreement of a comprehensive geriatric consultation, with the future interest in evaluating the performance of a brief assessment tool for use in the primary care context.

## Method

### Setting and population

This study was conducted at the outpatient clinic of the Service of Geriatric Medicine, University of Lausanne, Switzerland. All community-dwelling older persons aged 70 years and over referred by their primary care physician to this clinic for a comprehensive geriatric assessment were eligible, and they were asked to participate to the study. In Switzerland, geriatric departments of hospitals can also provide ambulatory geriatric consultations for old patients referred by GP’s. Patients were excluded if aged less than 70 years, if they hadn’t a primary care physician (e. g. patients referred directly to the geriatric consultation after an emergency department visit), if they were unable to speak French, or if they were already assessed by a geriatrician within the last 12 months. The aim of the latter exclusion criterion is to avoid the possible bias in reliability estimation due to the availability of recent geriatric information about the patient. Finally, initially enrolled patients were excluded if a major medical event occurred in-between the two geriatric consultations (e.g. hospitalization). For this descriptive study, a convenient sample of 20 participants was obtained.

### Geriatric consultation

Each patient included in the study was seen at two different times at less than 2 months distance and assessed by two different geriatricians randomly selected among nine trained graduated geriatricians from the Service of Geriatric Medicine. Geriatricians were blinded to the scores and conclusions of the other patient’s visit. This consultation was based on a structured evaluation process aimed to determine health problems in older patients, to identify their resources and needs for care and services. This structured approach was based on the use of a combination of validated screening tests and geriatrician’s clinical judgment. This consultation lasted about 2 h, which corresponds to the usual duration of a geriatric consultation in our setting. Domains assessed during the geriatric consultation and corresponding instruments are presented in Additional file 1: Table S1. All scores used such as MMSE or Katz’s instruments, are validated instrument and routinely used in geriatric consultation [13]. Practically, all tests and scores were performed systematically in the same way following a written check-list (as described in Additional file 1: Table S1). Cut-offs for the interpretation of the different tests are those usually provided by the authors and found in the literature. This objective evaluation was complemented by a clinical assessment performed by the geriatrician who could do additional medical history taking and health status checking to specify certain elements that were unclear during the first part of the evaluation. This is the plus-value of this complementary approach combining objective testing and patient-centered clinical expert judgment. All geriatricians working in the department (and participating to this study) were trained to follow this standardized consultation process.

### Data collection

During each of the two consultations, the results of the following tests were systematically collected: Basic and Instrumental Activities of Daily Living (ADL) [13, 14], Mini Mental Status Exam (MMSE), Clock Drawing Test [15, 16], Geriatric Depression Scale (GDS) [17, 18], Mini Nutritional Assessment (MNA) and Performance Oriented Mobility Assessment (POMA) status [19]. The following at-risk medications were also recorded: anticholinergics, antihistaminics, psychotropes (tricyclics antidepressants, benzodiazepines, neuroleptics) and non steroid anti-inflammatory drugs. At the end of each consultation, the following geriatric conditions were systematically rated (in 2 or 3 categories) and collected: functional and cognitive impairment, mood disorders, risk of fall, osteoporosis; malnutrition, urinary incontinence, visual and hearing impairment (Table 1). Categorization for each syndrome in one of the rating categories was left to the geriatrician’s judgment. Geriatricians based their evaluation on results of tests complemented by their expert clinical assessment of patient health status. At the end of the consultation, geriatricians were asked to rate the patient’s overall health status, choosing among “Robust”, “Vulnerable”, and “Dependent”. This rating was based on the results of all tests and the clinical judgment of the geriatricians.

**Table 1:** Geriatric conditions assessed by the geriatrician and likelihood rating and prevalence of disorders at the first visit

Condition	Possible categories	Dichotomization	Prevalence at the first visit
Functional impairment	Severe/ Mild / Moderate	Limited = 0	47%
		Mild/Important = 1	
Cognitive impairment	Ascertained / Possible / Absent	Absent = 0	75%
		Possible/ Ascertained = 1	
Mood disorders	Ascertained / Possible / Absent	Absent = 0	60%
		Possible/ Ascertained = 1	
Risk of fall	High / Moderate / low	Low = 0	65%
		Moderate/High = 1	
Osteoporosis	Yes / No	No = 0	3%
		Yes = 1	
Malnutrition	Malnutrition / at risk of malnutrition / absence of malnutrition	Absence of malnutrition = 0	40%
		At risk of malnutrition/malnutrition = 1	
Incontinence	Urgency / Stress/ Mixed / No incontinence	No incontinence = 0	55%
		Urgency/Stress/Mixed = 1	
Visual impairment	Severe / moderate / absence of impairment	Absence of impairment = 0	90%
		Moderate/ Severe = 1	
Hearing impairment	Severe / moderate / absence of impairment	Absence of impairment = 0	65%
		Moderate/ Severe = 1	

Data were collected on paper or on electronic questionnaires (DOC-R application for Smartphone developed by ICT®, Switzerland). All data were entered on an Excel Spreadsheet and double checked by an independent research collaborator.

### Statistical analyses

Categorical answers were grouped in a dichotomous way, by opposing the absence of a condition to a certain likelihood of this condition (Table 1). For each geriatric condition, reliability and agreement of the binary answer were assessed by means of three-way Intraclass Correlation (3w-ICC) [11, 12] and three-way Observed Disagreement (3w-OD). The latter can be decomposed into disagreements due to each source of error variability: visit, rater and random. Both three-way indexes were compared with their two-way counterparts (2w-ICC and 2w-OD), the latter two approaching the traditional Kappa and Observed Disagreement, respectively, when only the visit effect is taken into account. Confidence intervals around two-way and three-way indexes were estimated using a simulation-based approach [20] and assuming a multivariate log-normal distribution of the variance component in the random effect model. Additional file 2 contains technical details of each quantity definition. A simulation was also performed in order to identify eventual bias on small samples of the two-ways quantities 2w-ICC and 2w-OD, when several sources of error variability do exist into simulated data. Statistical analyses were performed using R software package, version 3.2.2 (R: A language and environment for statistical computing. R Foundation for Statistical Computing, Vienna, Austria, URL: http://www.R-project.org), library “lme4”, function lmer(), Maximum Likelihood estimation method.

## Results

Main characteristics of the population are described in Table 2, along with the median [IQ-range] scores of the quantitative tests described in the method section and Additional file 1: Table S1. The median time between the two consultations was 18 days (IQR: 10–37). Median duration was 105 min for the first consultation (range: 75–150 min) and 90 min for the second (range: 60–190). 40% of patients were taking at-risk medication at the first consultation; 25% of patients were rated as robust, 45% vulnerable and 30% dependent. No major change in health status was observed among the included patients between the two visits.

**Table 2:** Socio-demographic and functional characteristics of the study population (*N* = 20) as assessed in the first consultation

Patients characteristics	(*n* = 20)
Age (Mean ± SD)	80.8 ± 8.0
Women (N[%])	7[35]
Foreign citizenship (N[%])	6[30]
Basic ADL^a^ (Median [IQ range])	5 [5–6]
Instrumental ADL^b^ (Median [IQ range])	5 [3–7]
MMSE Score^c^ (Median [IQ range])	28 [26–29]
MMSE < 24 (N[%])	3[15]
Clock-drawing test^d^ (Median [IQ range])	10 [8–10]
GDS Score^e^ (Median [IQ range])	3.5 [2–5.5]
Tinetti POMA Score^f^ (Median [IQ range])	26 [21.5–27]
Number of patients with at risk medications^g^ (N[%])	8[40]
Health status (N[%])	
Robust	5[25]
Vulnerable	9[45]
Dependent	6[30]

^a^Katz’s basic activities of daily living (ADL) [13]: score from 0 to 6, higher score indicating greater independence.

^b^Lawton’s instrumental ADL: score from 0 to 8, higher score indicating greater independence.

^c^Folstein’s Mini Mental State Examination [15]: score from 0 to 30, higher score indicating better cognition.

^d^CDTest range was 0 to 10^.^

^e^Yesavage’s Geriatric Depression Scale [17, 18]: score from 0 to 15, higher scores indicating higher depressive symptoms.

^f^Tinetti’s Performance Oriented Mobility Assessment [19]. score from 0 to 28, higher score indicating higher gait and balance performance.

^g^At risk medication categories: Tricyclic antidepressants, antihistaminic, anticholinergic, anti-inflammatory drugs, hypnotic, neuroleptic and others.

Table 3 provides agreement and reliability estimates concerning items described in Table 1. For each item a two-way estimate of ICC (2w-ICC) and OD (2w-OD) is compared with the three-way estimate of the same quantities (3w-ICC and 3w-ICC). OD estimates arising from the three-way model were similar to the ones obtained with the two way model for 4 geriatric conditions (functional/cognitive/visual impairment, and incontinence), they were larger for 3 geriatric conditions (mood disorders, risk of fall, and osteoporosis signs), and they were smaller in 2 instances (malnutrition and hearing impairment). The same observation (but in the opposite direction) arises when comparing the 3w-ICC estimate with the 2w-ICC estimate, the latter approximating the kappa.

**Table 3:** Intraclass Correlation (ICC) and Observed Disagreement (OD) estimated on 9 Items using two and three-way random effect models

		Two-way random effect model									Three-way random effect model									
	Kappa	Subj.	Time	Rand.	2w-OD	(95% CI)		2w-ICC	(95% CI)		Subj.	Time	Rater	Rand.	3w-OD	(95% CI)		3w-ICC	(95% CI)	
Functional impairment	0.68	0.34	0.00	0.16	0.16	*(0.08;*	*0.29)*	0.68	*(0.44;*	*0.85)*	0.34	0.00	0.00	0.16	0.16	*(0.08;*	*0.29)*	0.68	*(0.44;*	*0.85)*
Cognitive impairment	0.62	0.25	0.00	0.15	0.15	*(0.08;*	*0.27)*	0.62	*(0.37;*	*0.82)*	0.27	0.00	0.04	0.11	0.15	*(0.06;*	*0.44)*	0.64	*(0.31;*	*0.84)*
Mood disorders	0.80	0.40	0.004	0.10	0.10	*(0.05;*	*0.21)*	0.80	*(0.58;*	*0.91)*	0.27	0.004	0.04	0.10	0.15	*(0.05;*	*0.21)*	0.80	*(0.58;*	*0.91)*
Risk of fall	0.68	0.32	0.00	0.15	0.15	*(0.08;*	*0.27)*	0.68	*(0.44;*	*0.85)*	0.18	0.00	0.13	0.11	0.24	*(0.11;*	*0.61)*	0.42	*(0.15;*	*0.71)*
Osteoporosis	0.68	0.33	0.00	0.16	0.16	*(0.08;*	*0.29)*	0.68	*(0.43;*	*0.85)*	0.28	0.00	0.05	0.13	0.18	*(0.08;*	*0.49)*	0.61	*(0.27;*	*0.83)*
Malnutrition	0.00	0.00	0.00	0.44	0.44	*(0.28;*	*0.67)*	0.00	*(0.00;*	*0.00)*	0.06	0.00	0.16	0.22	0.38	*(0.19;*	*0.79)*	0.14	*(0.01;*	*0.53)*
Incontinence	0.70	0.35	0.00	0.15	0.15	*(0.08;*	*0.27)*	0.67	*(0.46;*	*0.86)*	0.35	0.00	0.00	0.15	0.15	*(0.08;*	*0.27)*	0.70	*(0.46;*	*0.86)*
Visual impairment	-0.11	0.00	0.00	0.18	0.18	*(0.12;*	*0.28)*	0.00	*(0.00;*	*0.00)*	0.00	0.00	0.00	0.18	0.18	*(0.12;*	*0.28)*	0.00	*(0.00;*	*0.00)*
Hearing impairment	0.56	0.26	0.00	0.20	0.20	*(0.10;*	*0.36)*	0.56	*(0.30;*	*0.78)*	0.28	0.00	0.03	0.16	0.19	*(0.09;*	*0.40)*	0.60	*(0.32;*	*0.81)*

With a simulation study we showed that, when a visit and a rater effect are both present into the data, the two-way model systematically overestimates reliability and underestimates disagreement, while results are unbiased when using the three-way model, even in very small samples (results not shown). The three-way model estimated a disagreement ranging between 15% and 38% (agreement between 62% and 85%, mean agreement 81%). The proportion of disagreement due to the visit effect was none, except for mood disorders, where time change explained 3% of the total disagreement (=0.004/0.15, mood disorders, Table 3). Error variability due to geriatrician explained a proportion of disagreement ranging from 0% (functional/visual impairment, and incontinence) to 54% = 0.13/0.24 (risk of fall, Table 3). Reliability ranged between 0 and 80% (mean 3w-ICC 51%). Referring to the reliability classification introduced by Altman [21], reliability was *poor* for visual impairment and malnutrition (3w-ICC < 0.2); *moderate* for assessments of risk of fall (0.4 ≤ 3w-ICC < 0.6), and *good* for functional/cognitive/hearing impairment, osteoporosis, and incontinence assessments (0.6 ≤ 3w-ICC < 0.8). Reliability was *excellent* for mood assessment (3w-ICC ≥ 0.8).

## Discussion

### Clinical significance of the results

No studies investigating reliability of a complex clinical consultation was found in the literature (e.i., taking into account numerous clinical domains). Indeed, often agreement or reliability assessments focused on one specific item. To investigate reliability of a comprehensive consultation as the one provided by geriatricians is however important, because the latter represents a reference consultation for primary care physicians and other care providers, and it offers the basis for initiating management options. In this perspective the current work represents a rare occasion to provide information on how reproducible a geriatric consultation is in real life setting.

As each geriatric condition was assessed by specific tests, no attempt was made to define an overall agreement score or index for the entire consultation, but rather to assess agreement and reliability for each condition. This study showed that the reliability of a geriatric consultation can be considered as good to excellent (3w-ICC ≥ 0.6) for six out of nine geriatric conditions (functional/cognitive/hearing impairment, osteoporosis, incontinence, and mood disorders). In contrast, reliability was only moderate (3w-ICC ≥ 0.4) for one geriatric condition (risk fall), and poor (3w-ICC < 0.2) for two conditions (visual impairment and malnutrition).

In the case of malnutrition, the low reliability estimate was essentially related to the very low observed agreement (almost half of patients were differently classified at the two visits). This result is not surprising considering that there is no consensual definition of malnutrition or standardized instruments to measure it. Furthermore malnutrition identification relies on the assessment of medical history by inquiring if patient has lost weight over a certain period of time, or if his or her caloric intake is diminished. It is therefore likely that older patients might not know their weight and thus estimating quantitative loss of weight over time is rather hazardous, as well as for quantifying intakes. Another explanation is that the Mini-Nutrition Assessment (MNA) score might have been inconsistently done by geriatricians and was then potentially only seldom used to assess overall malnutrition. The use of the MNA short form (MNA-SF) might have improved the overall assessment of malnutrition through a better adherence. This might also explain the poor agreement and reliability. Finally it is also likely that primary care physicians who are following patient over long period of time will be able to better estimate recent weight loss by using records of their medical file. Assessment of malnutrition by geriatricians would be improved if electronic data about the patient weight history were available, and an objective weight loss calculation could replace the subjective reporting of the patient.

The case of visual impairment is a different one: reliability was low despite that agreement was good (80% of patients were equally classified at the two visits). The reason of this apparently contradictory result should be found in the very low between-subject variability observed for visual impairments: at each visit 90% of subjects were classified as having visual impairments. Reliability indexes (kappa, ICC) being defined as the percentage of between-subject variability over the total variability, it is not surprising that their value is low when individuals are very similar of each other, despite the high agreement between visits/raters. The reliability - and also the significance - of this measure would be improved if an evaluation of visual corrections (glasses) adopted by the patient replaced the simple assessment of his/her visual performance.

Reliability of risk of fall (only moderate) probably suffers from the same subjectivity of the patient reporting. Indeed assessing the risk of fall relies mainly on past history of falls. Having access to full electronic health records might limit this important recall bias. Furthermore, more objective tests such as timed up and go have proven to be of limited value in assessing the risk of fall [22].

Reliability of mood disorder assessment was excellent. This is probably due to the fact that it relies on highly standardized detailed scoring scale (Yesavage’s Geriatric Depression Scale) that leaves little room for subjective interpretation by the rater. Similarly, good results for the assessment of functional impairment and cognitive impairment were also obtained that might also be explained by the fact that both rely on detailed scoring scales. Finally, even though a full geriatric assessment as described here can act as a reference consultation for older patients, it seems not feasible that the entire population of older people could benefit from it. Rather, collaboration with primary care physicians who screen for common geriatric syndromes might be a more judicious approach.

### Methodological interest

The approach described in this study showed that using 3-way intraclass correlation (3w-ICC) index performs better than usual Kappa test to assess the reliability, when different sources of error variability are present in the data. It allows indeed calculating the disagreement attributable to each source of variability (observed disagreement decomposition) and avoids biased results on small samples, as shown by a simulation. On geriatric consultations data, assessment of reliability using 3w-ICC index provided slightly different and more accurate measurement with respect to the classical Kappa analysis, showing notably that reliability on the assessment of fall risk and global health status is lower (and the disagreement is higher) than when estimated with the traditional Kappa test. Furthermore, we estimated that variability related to the geriatrician related error plays an important role in the overall disagreement, while error variability related to the visit effect is almost inexistent (with 1 month distance visits), contrary to what one could expect when dealing with older people.

### Limitations and strengths

This study has some limitations. Fist, the small sample size might have impacted on the results. Reliability and agreement are estimated correctly by means of 3-way quantities 3w-ICC and 3w-OD (as shown by simulation), but with a large variability. This is reflected by confidence intervals that often cover two or three categories of the Altman’s reliability classification (poor / fair / moderate / good / excellent). Second, by grouping categorical answers in a dichotomous way, we may have modified slightly the true agreement. With our large definition of disease as the *potential* presence of a problem a negative test will lead to reasonably exclude the disease for a patient, but a positive result will not confirm the diagnosis instead requiring further investigation. Last, the accuracy and performance of the assessment was not tested in the present study, whose aim was rather to insure that each geriatrician performing the assessment will come up with the same results for the same patient.

The study has also strengths. For the first time the reliability of a comprehensive geriatric consultation including several aspects of an elder’s health was analyzed. In addition, the in-depth methodological approach (3-way measures) allowed to distinguish between different sources of disagreement for each geriatric measure, and to specifically estimate disagreement attributable to raters’ variability.

## Conclusions

Overall, the reliability of the geriatric consultation can be considered as good. It could be further improved by a more consistent use of standardized tests, in particular for malnutrition assessment. Such a consultation can be used confidently as a reference consultation by other care providers such as GP physicians for selected patients. Indeed, it seems important that basic screening for common geriatric syndromes is performed in first line by GP’s and only complex cases referred to the specialist. It could also serve as a gold standard for clinical research projects when a comprehensive geriatric assessment is foreseen.

## Additional files

Additional file 1: Table S1. Domains Investigated and Tests Used to Assess the Geriatric Patient. Domains assessed during the geriatric consultation and corresponding instruments. (DOCX 26 kb)

Additional file 2: Two and three-way Intraclass Correlation Coefficient and Observed Disagreement. Technical details about statistics used in the study. (DOCX 66 kb)
