# Supplementary material for: A prospective study assessing agreement and reliability of a geriatric evaluation
Source: BMC Geriatr. 2017 Jul 19;17:153. doi: 10.1186/s12877-017-0546-9 (PMC5517926; doi:10.1186/s12877-017-0546-9)
Supplement: Supplementary file 1 — Domains Investigated and Tests Used to Assess the Geriatric Patient. Domains assessed during the geriatric consultation and corresponding instruments. (DOCX 26 kb) [file 12877_2017_546_MOESM1_ESM.docx]

**Additional file 1: Table S1: Domains Investigated and Tests Used to Assess the Geriatric Patient**

| Functional impairment | Katz’s basic activities of daily living (ADL)^1^ : score from 0 to 6, higher score indicating better functional status  Lawton’s instrumental ADL^2 3^: score from 0 to 8, higher score indicating greater independence |
| --- | --- |
| Cognitive impairment | Folstein’s Mini Mental State Examination^4^: score from 0 to 30, higher score indicating better cognitive function  Reported cut-off: 24-19= mild, 18-10= moderate and < 10= severe  Clock Drawing Test^5 6^ : score from 0 to 10, higher score indicating better cognitive function. Reported cut-off for increased risk of cognitive impairment: 8 |
| Mood disorders | Yesavage’s Geriatric Depression Scale^7^: score from 0 to 15, higher scores indicating higher depressive symptoms. Reported cut-off for likely depression: 6 |
| Risk of fall | Performance Oriented Mobility Assessment^8 9^: score from 0 to 28, higher score indicating higher gait and balance performance. Reported cut-off for increased risk of fall: < 20  Fall history : no fall or ≥1 fall during the last year |
| Osteoporosis | Height loss of >2 cm for women and >3 cm for men.  Wall-Occiput Test >0 cm.  Rib-Pelvis Distance test ≤2 Fingerbreadths |
| Malnutrition | Loss of weight >5% within 1 months or >10% whithin 6 months.  Mini Nutritional Assessment (MNA)^10^  Body Mass Index |
| Incontinence | History^11^ |
| Visual impairment | Snellen scale^11^ |
| Hearing impairment | Whisper test^11^ |
| At risk medication | anticholinergics, antihistaminics, psychotrop(tricyclics antidepressants, benzodiazepines, neuroleptics) et non steroid anti-inflammatory drugs |

1. Katz S. Assessing self-maintenance: activities of daily living, mobility, and instrumental activities of daily living. J Am Geriatr Soc 1983;**31**(12):721-7.

2. Graf C. The Lawton Instrumental Activities of Daily Living Scale. AJN The American Journal of Nursing 2008;**108**(4):52-62 10.1097/01.NAJ.0000314810.46029.74.

3. Lawton MP, Casten R, Parmelee PA, et al. Psychometric characteristics of the minimum data set II: validity. Journal of the American Geriatrics Society 1998;**46**(6):736-44.

4. Folstein MF, Folstein SE, McHugh PR. "Mini-mental state". A practical method for grading the cognitive state of patients for the clinician. J Psychiatr Res 1975;**12**(3):189-98.

5. Borson S, Scanlan J, Brush M, et al. The Mini-Cog: a cognitive ‘vital signs’ measure for dementia screening in multi-lingual elderly. International Journal of Geriatric Psychiatry 2000;**15**(11):1021-27.

6. Milne A, Culverwell A, Guss R, et al. Screening for dementia in primary care: a review of the use, efficacy and quality of measures. International Psychogeriatrics 2008;**20**(05):911-26.

7. Yesavage JA. Geriatric Depression Scale. Psychopharmacology bulletin 1988;**24**(4):709-11.

8. Ganz Da BYSPGRLZ. WIll my patient fall? JAMA 2007;**297**(1):77-86.

9. Tinetti ME. Performance-oriented assessment of mobility problems in elderly patients. J Am Geriatr Soc 1986;**34**(2):119-26.

10. Vellas B, Guigoz Y, Garry PJ, et al. The mini nutritional assessment (MNA) and its use in grading the nutritional state of elderly patients. Nutrition (Burbank, Los Angeles County, Calif) 1999;**15**(2):116-22.

11. Senn N, Monod S. Development of a comprehensive approach for the early diagnosis of geriatric syndromes in general practice. Frontiers in Medicine 2015;**2**.
