# Supplementary material for: A prospective study assessing agreement and reliability of a geriatric evaluation
Source: BMC Geriatr. 2017 Jul 19;17:153. doi: 10.1186/s12877-017-0546-9 (PMC5517926; doi:10.1186/s12877-017-0546-9)
Supplement: Supplementary file 2 — Two and three-way Intraclass Correlation Coefficient and Observed Disagreement. Technical details about statistics used in the study. (DOCX 66 kb) [file 12877_2017_546_MOESM2_ESM.docx]

**Additional file 2**

**Two and three-way Intraclass Correlation Coefficient and Observed Disagreement**

We consider the case of *m* > 2 raters evaluating *n* subjects two different times (visit 1 / visit 2) on a dichotomous variable. Data can be summarized in a 2x2 table of the form:

| visit 1 / visit 2 | Present | Absent |
| --- | --- | --- |
| Present | *a* | *b* |
| Absent | *c* | *d* |

being $a,b,c$ and $d$ the observed number of subjects within each category, with $a+b+c+d=n$. A standard agreement/reliability analysis of the above data would consist in calculating the well-known kappa index^2^. Kappa is defined as follows: $\kappa=\left(Po-Pe\right)/\left(1-Pe\right)$, where $P_{O}$ represents the proportion of subjects equally classified at the two visits: $P_{O}=\left(a+d\right)/n$ (observed agreement), and $Pe$ represents the proportion of subjects who would be equally classified under the assumption of independence between the two visits: $Pe=((a+b)(a+c)+(b+d)(c+d))/n^{2}$ (chance agreement). Several authors underlined the asymptotic equivalence of the Kappa index with the variance decomposition arising from a two-way (subject + visit) random effect model of the form: $y_{ij}=\mu+s_{i}+v_{j}+e_{ij}\text{ }$(model M1). Here $y_{ij}$ is the score of individual $i$ at visit$j$, $\mu$ is a fixed parameter representing the global mean score, $s_{i}$ represents the subject effect, $v_{j}$ the visit effect and $e_{ij}$ the residual effect. The random effects $s_{i}$, $v_{j}$ and $e_{ij}$ are supposed to be independent and normally distributed, with variances equal to $\sigma_{s}^{2}$, $\sigma_{v}^{2}$ and $\sigma_{e}^{2}$, respectively. Let $\hat{\sigma}_{s}^{2}$, $\hat{\sigma}_{v}^{2}$ and $\hat{\sigma}_{e}^{2}$ be the model estimated variances. Fleiss and Cohen^1^ showed that: 1) The proportion of subject variability $\hat{\sigma}_{s}^{2}$ over the total variability approximates the kappa for *n* sufficiently large. This quantity defines another reliability index, the *Intraclass Correlation Coefficient, ICC*^2 3^ 2) The observed agreement $Po$ can also be obtained from (M1) using the *exact* relation: . The last relationship motivated us to define an Observed Disagreement (OD) index as follows: . This quantity can be decomposed into a disagreement due to the visit effect ($2\hat{\sigma}_{v}^{2}$), and a disagreement due to the residual random effect ($2\hat{\sigma}_{e}^{2}$). The Kappa index and the observed disagreement can thus be recovered via a two-way variance decomposition. However, the estimate of the observed disagreement fail to account for the presence of several raters involved in the evaluation process at each visit. It may be important to consider the possibility that a rater-related error is contained into the data. In order to do this, a third random effect (a *rate*r effect) should be introduced into model (M1), thus becoming a three-way (subject + visit + rater) random effect model:$y_{ijk}=\mu+s_{i}+v_{j}+r_{k}+e_{ijk}\text{ }$(model M2). Here $y_{ijk}$ is the score of individual $i$ at visit $j$ from rater $k$, $r_{k}$ represents the rater effect, supposed to be independent from the other random effects and also normally distributed, with variance equal to $\sigma_{r}^{2}$. According to (M2) one can define a three-way Intraclass Correlation Coefficient (3w-ICC) and a three-way Observed Disagreement (3w-OD), taking into account both the rater and visit variability:and , being $\hat{\sigma}_{s}^{2}$, $\hat{\sigma}_{v}^{2}$, $\hat{\sigma}_{r}^{2}$ and $\hat{\sigma}_{e}^{2}$ estimated variances arising from model (M2).

---------------------------------------------------------------------------------------------------------------------

1. Fleiss JL, Cohen J. The equivalence of weighted kappa and the intraclass correlation coefficient as a measure of reliability. Eucational and Psychological Measurement 1973;**33**(613-19).

2. Bartko JJ. The intraclass correlation as a measure of reliability. Psychological reports 1966;**19**(3-11).

3. Rousson V, Gasser T, Seifert B. Assessing intrarater, interrater and test-retest reliability of continuous measurement. Statistics in Medicine 2002;**21**(3431-46).
